# A prospective study of cumulative job stress in relation to mental health

**DOI:** 10.1186/1471-2458-5-67

**Published:** 2005-06-15

**Authors:** Isabelle Godin, France Kittel, Yves Coppieters, Johannes Siegrist

**Affiliations:** 1 School of Public Health Université Libre de Bruxelles, HealthPsychology Unit CP 596, 808 Route de Lennik, 1070 Brussels, Belgium; 2 School of Public Health Université Libre de Bruxelles, Epidemiology Department, 808 Route de Lennik, 1070 Brussels, Belgium; 3 Department of Medical Sociology, University of Duesseldorf, Universitaetsstr. 1, 40225 Duesseldorf, Germany

## Abstract

**Background:**

This study tests associations between psychosocial stress at work measured by the effort-reward imbalance model in a dynamic perspective, and multiple indicators of poor mental health, in a prospective design.

**Methods:**

1986 male and female employees from four Belgian enterprises were followed-up over one year within the framework of the Somstress study. Based on two consecutive measurements, an index of cumulative job stress was constructed and its associations with five indicators of mental health were studied, excluding caseness at entry (for depression, anxiety, somatisation, chronic fatigue and psychotropic drug consumption respectively). Taking into account the longitudinal design, four categories of job stress are defined: 1) employees free from stress at both measures, 2) job stress present at first measure but not at the second one, 3) recent onset of job stress as evidenced by second measure 4) workers exposed to stress at both measures. Multivariate logistic regression with appropriate adjustments was applied.

**Results:**

In bivariate analysis, a clear graded association of cumulative job stress with all five mental health indicators is observed, both in men and women. In multivariate logistic regression analysis, recent onset of stress is strongly associated with poor mental health among men (odds ratios ranging from 1.8 to 4.6), while cumulative stress shows strongest effects on mental health in women (odds ratios ranging from 1.4 to 7.1).

**Conclusion:**

Cumulative experience and recent onset of job stress in terms of high effort spent and low reward received is associated with elevated risk of all five indicators of poor mental health at follow-up in a large cohort of employees.

## Background

Instability of employment, rapid change of demands and intensification of work pressure are widely prevalent consequences of economic globalization and technological change [1]. Even in established sectors of industrial production, administration and services of advanced societies experiences of downsizing, mergers and outsourcing are increasingly shared by employees [2]. Surveys of working conditions in Europe indicate that stressful experience recently increased in the European workforce although variations between countries and sectors are observed [3]. Chronic stressful experience at work can adversely affect physical and mental health. This has been documented in a large number of epidemiological studies based mainly on two complementary theoretical concepts, the demand-control model [4-6] and the effort-reward imbalance model [[7,8]; see also [9-12]]. The demand-control model posits that jobs characterised by high quantitative demands in combination with low decision latitude adversely affect health. The focus of the effort-reward imbalance model is put on contractual non-reciprocity where high efforts at work are not met by adequate rewards in terms of money, esteem, promotion prospects and job security.

Both models have been tested in the frame of prospective epidemiological studies, but in a majority of these investigations the measurement of exposure was restricted to baseline assessment. Thus, effects of cumulative job stress on incident disease have not been sufficiently explored so far. However, with regard to the demand-control model, there are a few important exceptions indicating, firstly, that recurrent job stress is indeed associated with elevated risk of ill health [13,14], morbidity [15,16], and mortality [17], and, secondly, that reduction of job stress over time results in improved health [18]. No comparable data on health effects of cumulative stress are available from the effort-reward imbalance model. This is particularly critical as this model documents close links with macroeconomic changes, suggesting that downsizing, mergers and outsourcing result in increases of effort-reward imbalance [19] and, thus, may indirectly affect health.

This study analyses the dynamics of stressful work experience over time, based on the effort-reward imbalance model, in relation to mental health, using longitudinal data of a large cohort. We test the hypothesis that the risks of poor mental health after one year are higher among employees who either continuously experience high job stress or who experience an increase in job stress from the first to the second measurement, compared to the remaining employees with either continuously low levels or decreasing levels of job stress over time. Both conditions, continuous exposure and incident exposure to job stress, are more likely to occur under conditions of downsizing and related macroeconomic constraints.

For two additional reasons, the effort-reward imbalance model is chosen to measure stressful experience at work. First, as mentioned, two of the three reward components specified in this model provide a direct link with the labour market dynamics that are becoming increasingly relevant in a globalized economy: promotion prospects including job security and level of salary or wage [20]. In addition the model is composed by an extrinsic component (perceived demands, perceived rewards) and an intrinsic component (coping with demands at work; overcommitment as a motivational risk factor), thus allowing for a differentiation of 'subjectively perceived' situational and personal characteristics of stressful experience at work. Secondly, the standard measurement of this model, a Likert-scaled self-administered questionnaire (see Methods) was shown to be highly sensitive to change of exposure over time [21]. This is an essential prerequisite of studying dynamics of stressful experience at work in a reliable way.

## Methods

### Study design

Somstress is a Belgian research project based on a prospective protocol with a repeated measure of exactly the same self-administered questionnaire in a one year time interval. Its main purpose is to assess mental health and psychological well-being in relation to working conditions by combining organizational, psychosocial and behavioral data. Depression, anxiety, somatisation, chronic fatigue and, as a more objective correlate, psychotropic drug consumption, were chosen as indicators of mental health according to the research protocol. A previous publication restricted to cross-sectional baseline data already documented associations of psychosocial stress at work with these indicators of mental health [22]. Yet, the current report is the first to analyse these associations prospectively.

### Study sample

Four enterprises were selected according to their economic stability in order to include contextual variation into the study design. To this end, a special index of economic instability was constructed (for description see [23,24]). This index reflects the extent and change over the past three years in the employment and unemployment rates in each one of a broad spectrum of economic activities, based on a national coding system. A larger number of companies was evaluated accordingly. In the final selection process, four enterprises were included. The four selected companies differ gradually on this variable, ranging from a very stable company (Firm 1) to a very unstable company (Firm 4). All four companies belong to the private or public service sector where the majority of employees are white collars.

At time 1 (T1, 2000) as at time 2 (T2, 2001), all workers of the four enterprises were invited to participate in the study. Participation was voluntary. At baseline, 9634 questionnaires were sent out, corresponding to the total number of workers, and 3804 were returned (global participation rate 40 per cent). A similar participation rate was achieved at follow up (T2) with 2709 questionnaires returned (global participation rate 37 per cent). However, full data from identical subjects obtained from both surveys was restricted to 1986 employees (paired sample). Compared to other studies, this participation rate is relatively low, and could be partially explained by the uncertainty and the feeling of threat induced by the possibility of merging and downsizing in several workplaces.

At the end of the study, 3 different combinations of samples of workers can be analyzed: all participants to the first measure (N = 3804), all participants to the second measure (N = 2707), and participants to both measures (N = 1986). In this paper, it is this last sample (paired sample) that will be analyzed because of its prospective properties and therefore the possibility of testing the study hypothesis. This sub-sample is well comparable with the larger sample (N = 3804 and 2709 respectively) in terms of major socio-demographic characteristics, such as age, sex, educational level and professional qualification.

Participants to the first measure only are very similar to the participants to both measures (paired sample). In other terms, those who were lost during the follow up do not differ in terms of socio-economic, demographic conditions or health status (self-rated health). We can therefore exclude a bias due to selective attrition. Moreover, both populations are representative of the whole population of workers, in each enterprise, for the available criteria: gender, age, occupation and department or service.

### Data collection

The fully standardized questionnaires contain data on socio-demographic characteristics of the respondent, on psychosocial stress at work and on indicators of mental health and psychological well-being (at T1 and T2). The same questionnaire was submitted at both measures (T1 and T2).

Effort-reward imbalance at work was measured by the original questionnaire [20] containing the three scales 'effort' (5 Likert scaled items; the 6th item measuring physical load was omitted as there were mainly white collars), 'reward' (11 Likert scaled items with three subscales 'esteem', 'salary and promotion prospects' and 'job security') and 'overcommitment' (short version with 6 Likert scaled items defining a one-dimensional scale). A score reflecting the extent of imbalance was constructed by a ratio of the two scales 'effort' (nominator) and 'reward' (denominator, adjusted for unequal number of items by a correction factor). In this study, as in other reports (e.g. [25]), the upper quartile of the distribution of the ratio defines the risk condition of chronic psychosocial stress at work. Similarly, a group at risk in terms of the intrinsic component of the model, overcommitment, was defined by scores in the upper tertile of the respective scale, according to the established procedure [8,20].

In order to evaluate the dynamics of stressful experience at work over time, the sample was divided into four groups based on values of a summary variable, the ratio between effort and reward scores: group 1 (the reference group) was composed of employees who were free from job stress at either occasion (scores on the ratio were lower than those in the upper quartile); in group 2, job stress was present at first, but no longer at second measurement; conversely, group 3 was characterized by an absence of job stress at first, but a demonstration of it at second measurement; finally, group 4 was composed of employees who continuously reported a high level of job stress at either occasion (for group description see Table 2). It is important to note that about 25 percent of the sample are considered at risk (group 3 and 4) in terms of our research hypothesis.

**Table 2:** Sociodemographic indicators of the four job stress groups

	Effort-reward imbalance at work (ERI) N (%)			
	T_1 _no-T_2 _no	T_1 _yes-T_2_no	T_1 _no-T_2 _yes	T_1 _yes-T_2 _yes

Sex (ns)				
Men	672 (64.7)	108 (10.4)	91 (8.8)	168 (16.2)
Women	561 (65.5)	88 (10.3)	95 (11.1)	112 (13.1)
Education (**)				
Lowest (vocational school or less)	416 (67.4)	60 (9.7)	43 (7.0)	98 (15.9)
Secondary school	222 (67.9)	31 (9.5)	24 (7.3)	50 (15.3)
College	409 (62.5)	81 (12.4)	83 (12.7)	81 (12.4)
Highest (university)	172 (63.9)	19 (7.1)	32 (11.9)	46 (17.1)
Age (ns)				
18–34 yrs	309 (67.9)	52 (11.4)	45 (9.9)	49 (10.8)
35–49 yrs	745 (63.7)	116 (9.9)	120 (10.3)	189 (16.2)
50 yrs and +	157 (66.2)	22 (9.3)	19 (8.0)	39 (16.5)

* p < 0.05, ** p < 0.01, *** p < 0.001

In addition to this model, job dissatisfaction (5 items) and threat perceived from global economy (3 items) were assessed using respective items from the Job Content Questionnaire [26].

We used validated mental health measures derived from the Symptom Check List SCL90 [27] for (1) depression (16 items), (2) anxiety (10 items) and (3) somatisation (12 items). Each one of these mental health indicators represents a distinct, psychometrically tested scale [22,27].

Chronic fatigue was included as a further indicator of impaired mental health as assessed by a 4-item-scale that was developed in a Dutch study [28]. Internal consistencies of the SCL90 scales give the following Cronbach's alpha values: 0.93 (T1 and T2) for depression, 0.86 (T1) and 0.89 (T2) for anxiety, and 0.86 (T1) and 0.87 (T2) for somatisation. For chronic fatigue, the respective value is 0.86.

In addition, a more objective correlate of mental health problems, amount of psychotropic drug consumption was assessed by a scale summarizing type and frequency of the consumption of tranquillizers, antidepressants and/or sleeping tablets during the last 4 weeks [29].

The description of the SCL90 depression variable gives a range of 58 (min. 16, max. 74) and a mean score of 24.2 (9.9 st. dev.). Those values are for anxiety: range 35 (min. 10, max. 45), mean 14.6 (5.8 st. dev.) and for somatisation: range 45 (min. 12, max. 57), mean 18.9 (7.0 st. dev.). For chronic fatigue, the range is 24 (min. 4, max. 28), and the mean 14.8 (6.7 st. dev.). Intercorrelations T1-T2 for the SCL90 scores give r^2 ^values of 45 per cent for depression, 40 per cent for somatisation and 48 per cent for anxiety.

Due to non-normal distribution of core variables we applied logistic instead of linear regression analysis. In order to identify groups at risk of impaired mental health, all mental health indicators were dichotomized at the upper quartile of each score distribution [22]. While we are aware of the loss of information due to dichotomization of core variables, we nevertheless maintain that the statistical models applied and the large sample size may give us conservative estimates of the hypothesized associations.

### Statistical analysis

Data analysis is mainly based on the longitudinal aspects, i.e. on the sample of 1986 participants with complete data from both surveys. We apply Mc Nemar tests for paired sample in order to test the evolution between the two measures (univariate analysis). Identification of predictors of mental health problems at T2 is done by logistic regression analysis.

The two components of the effort-reward imbalance model (effort-reward ratio and overcommitment) are introduced separately in order to assess their relative contribution to the estimation of mental health problems. In this study, further refined analyses are not conducted, such as a test of possible effect modification (e.g. degree of job instability, socio-economic status) in order not to loose statistical power. Rather, these variables, together with age, job dissatisfaction and threat from global economy are introduced as confounders into the multivariate analysis. All models are calculated separately for men and women and for the five mental health indicators. Interactions between independent variables were tested and a significant interaction between stress and gender was found, implicating separate analysis for gender.

Because people and work characteristics vary quite a lot across workplaces, this latter variable could play a confounding role, and, as such, was introduced in the multivariate analysis.

To measure incident mental health problems (onset of new cases), individuals presenting at T1 the mental health problem studied at T2 were excluded from the analysis. This was done for obvious methodological reasons although the overall sample size was reduced by some 25 per cent. As a consequence, about 75 per cent of the total paired sample will be included in each logistic regression model.

## Results

Socio-demographic, psychosocial and health-related characteristics of the sample are shown in Table 1, separately for the four enterprises. As specified above, the different combinations of sub-samples (all participants to T1, all participants to T2 and the paired sample, i.e. participants to both measures) do not differ in terms of sex, age, education, professional qualification as they are very similar in their mental health indicators.

**Table 1:** Sample description (paired sample, N = 1986)

	Enterprise 1 N (%)	Enterprise 2 N (%)	Enterprise 3 N (%)	Enterprise 4 N (%)	Total N (%)
Sex (***)					
Men	131 (23.9)	206 (42.8)	200 (68.5)	529 (79.7)	1066 (53.7)
Women	418 (76.1)	275 (47.2)	92 (31.5)	135 (20.3)	920 (46.3)
Education (***)					
Lowest (vocational school or less)	93 (17.0)	160 (33.7)	61 (21.0)	412 (64.1)	726 (37.1)
Secondary school	35 (6.4)	58 (12.2)	58 (19.9)	121 (18.8)	272 (13.9)
College	277 (50.5)	174 (36.6)	125 (43.0)	105 (16.3)	681 (34.8)
Highest (university)	143 (26.1)	83 (17.5)	47 (16.2)	5 (0.8)	278 (14.2)
Age(***)					
*Mean (standard dev.)*	*38.9 (8.14)*	*39.1 (8.75)*	*39.6 (9.0)*	*43.2 (7.2)*	*40.5 (8.4)*
18–34 yrs	162 (29.5)	150 (31.2)	92 (31.5)	69 (11.0)	473 (24.2)
35–49 yrs	338 (61.6)	279 (58.0)	154 (52.7)	457 (72.7)	1228 (62.9)
50 and +	49 (8.9)	52 (10.8)	46 (15.8)	103 (16.4)	250 (12.8)
Effort-reward imbalance (*)					
T_1 _no, T_2 _no	350 (68.2)	289 (63.2)	170 (60.3)	424 (65.9)	1233 (65.1)
T_1 _yes, T_2 _no	50 (25.5)	46 (10.1)	39 (13.8)	61 (9.5)	196 (10.3)
T_1 _no, T_2 _yes	56 (10.9)	55 (12.0)	27 (9.6)	48 (7.5)	186 (9.8)
T_1 _yes, T_2 _yes	57 (11.1)	67 (14.7)	46 (16.3)	110 (17.1)	280 (14.8)
Overcommitment (high) (**)	164 (30.1)	171 (35.9)	108 (37.2)	272 (41.1)	715 (36.3)
Health (highest quartiles)					
Depression (n.s.)	141 (26.0)	105 (22.0)	59 (20.3)	174 (26.5)	479 (24.3)
Anxiety (*)	125 (20.3)	127 (26.6)	55 (18.9)	178 (27.1)	485 (24.6)
Somatisation (**)	149 (27.4)	131 (27.3)	55 (18.9)	194 (29.6)	529 (26.9)
Chronic fatigue	141 (26.6)	132 (28.3)	74 (26.1)	183 (28.4)	530 (27.5)
Psychotropic drug consumption	119 (22.8)	91 (19.6)	43 (15.5)	162 (25.6)	415 (21.9)

* p < 0.05, ** p < 0.01, *** p < 0.001

While mean age of the sample is about 40 years and while about half of the sample is composed of women, we observe a considerable variation for those variables across the four enterprises. In particular, participants in enterprise 4 are significantly older and there are less women. In addition, their educational degree is also lower, and the rate of employees with poor health indicators is remarkably high compared to the other workplaces. These differences are partly explained by the fact that this latter enterprise is a telecommunication company which hires mainly (male) technicians and workers with low degree of qualification.

As mentioned, the dynamics of job stress are explored by defining four subgroups. The proportions of employees in each category of this summary variable are indicated in Table 2, together with socio-demographic characteristics. Besides education, a proxy measure of socioeconomic status, the groups did not differ with regard to these characteristics.

**Figure 1:**
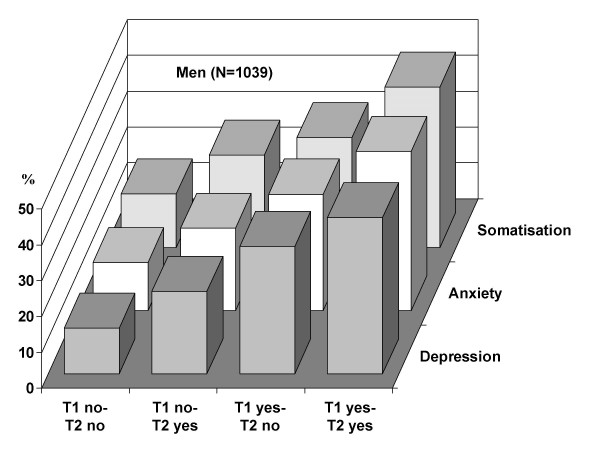
**Dynamics of stressful experience at work and prevalence of mental health (SCL90) at T2 (men). **For description of categories see text.

**Figure 2:**
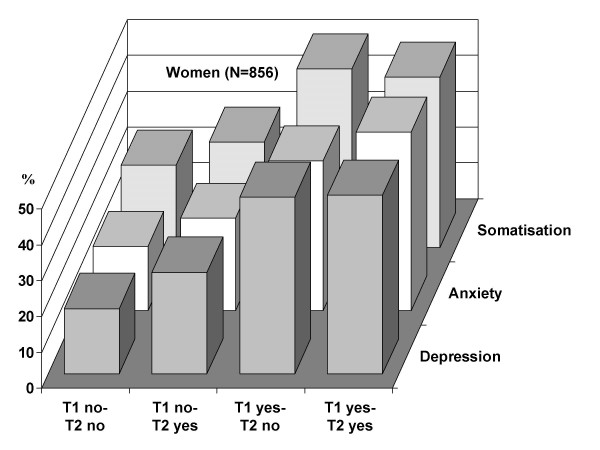
**Dynamics of stressful experience at work and prevalence of mental health (SCL90) at T2 (women). **For description of categories see text.

Relationships between the four job stress categories and mental health indicators show a steep gradient for depression, anxiety and somatisation, both in men and women (Figures 1 and 2). Less clear patterns were observed for chronic fatigue and psychotropic drug consumption (data not shown). Men and women reporting continuous or incident job stress during the observation period show higher proportions of mental health problems compared to those with low or decreasing job stress. Interestingly, the prevalence varies from about ten per cent in the group without stressful experience at work to about fifty per cent in the continuously stressed group, particularly among men.

As a final step, multivariate analysis is conducted to test the research hypothesis.

As indicated, logistic regression analysis is performed separately for men and women, with adjustment for age, education, threat from global economy, job dissatisfaction and work place instability. The dynamics of effort-reward imbalance at work over time are related to risks of poor mental health as suggested by our hypothesis. We do not observe a single effect in the group of employees who experienced a decrease in stressful work over time (group 2). In contrast, almost all odds ratios of mental health problems are significantly elevated in the two groups with newly emerging or continuously high job stress. In only 3 cases out of 20, odds ratios do not reach significance. There are interesting differences between gender. For men, recent onset of stressful experience at work is associated with a relatively highest risk of poor mental health, whereas for women relatively highest risks are observed in the group with continuously high job stress level for four indicators out of five. Odds ratios range between 1.4 and 7.1 for women and between 1.8 and 4.6 for men. When comparing the extrinsic versus the intrinsic component of the effort-reward imbalance model, it is obvious that associations of overcommitment with mental health are less consistent and generally weaker than those observed with the extrinsic component.

## Discussion

This study documents consistent associations of stressful experience at work over time with newly emerging mental health problems, using five mental health indicators. Employees with continuous job stress over a one year observation period and those with recently evolving job stress were at higher risk of developing poor mental health compared to the remaining groups, after exclusion of men and women with manifest mental problems at the study onset. One employee out of four in this large cohort belongs to one of the two groups with critical experience of job stress where, overall, a threefold elevated risk of incident poor mental health is observed. As the measurement of stressful experience at work is sensitive to change over time, it is unlikely that the observed systematic differences are spurious. Moreover, results remain true for men and women and after adjustment for relevant confounders, including the level of contextual job instability. While the gradient of mental health according to job stress is similar between men and women in bivariate analysis (Figure 1 and 2), an interesting gender difference results from multivariate analysis. Men are more reactive to a recent stress exposure, whereas women are more responsive to cumulative job stress (Table 3). This new finding needs further confirmation before being interpreted in a broader context.

**Table 3:** Poor mental health at T2 in relation to dynamics of stressful experience at work Multivariate logistic regression analysis (odds ratios (OR), 95% confidence intervals (CI))

men (N = 836): OR^$ ^(95% CI)					
	depression	anxiety	somatisation	chronic fatigue	psychotropic

Effort-reward imbalance^1^	***	**	**	**	**
T1 yes-T2 no	1.2 (0.5–2.9)	0.8 (0.3–2.1)	1.5 (0.7–3.4)	1.4 (0.7–3.1)	1.5 (0.6–4.1)
T1 no-T2 yes	4.6 (2.3–9.2)	3.7 (1.7–7.8)	4.1 (2.0–8.5)	3.4 (1.7–6.7)	3.2 (1.5–7.0)
T1 yes-T2 yes	2.8 (1.3–5.7)	2.3 (1.1–4.8)	2.0 (0.9–4.4)	1.8 (0.9–3.6)	3.4 (1.5–7.7)
Overcommitment	**	**	*	n.s.	n.s.
yes	2.4 (1.4–4.1)	2.5 (1.5–4.4)	1.8 (1.1–3.1)	1.2 (0.8–2.0)	1.1 (0.6–2.1)
women (N = 700): OR^$ ^(95% CI)					

	depression	anxiety	somatisation	chronic fatigue	psychotropic

Effort-reward imbalance^1^	***	**	**	***	*
T1 yes-T2 no	1.3 (0.5–3.2)	1.1 (0.4–3.1)	1.6 (0.5–4.5)	1.2 (0.5–3.1)	1.0 (0.3–3.0)
T1 no- T2 yes	3.2 (1.6–6.4)	2.3 (1.1–4.8)	3.5 (1.7–7.2)	2.0 (0.9–4.1)	2.7 (1.3–5.6)
T 1 yes-T2 yes	4.6 (2.3–9.0)	4.5 (2.1–9.8)	3.6 (1.6–8.2)	7.1 (3.4–14.5)	1.4 (0.5–3.5)
Overcommitment	*	n.s.	n.s.	n.s.	n.s
yes	1.8 (1.0–3.0)	1.6 (0.9–2.9)	0.8 (0.4–1.4)	1.1 (0.6–2.0)	1.4 (0.7–2.6)

^1^reference group: effort-reward imbalance T1 0, T2 0 : OR = 1.0

^$ ^adjusted for age, education, threat from global economy, job dissatisfaction, workplace instability

* p < 0.05, ** p < 0.01, *** p < 0.001

Despite this evidence, this study suffers from several limitations. First, although employees with manifest mental health problems at T1 were excluded from multivariate analysis, we cannot rule out the possibility that a deterioration of mental health during the observation period has affected the measurement of job stress at T2. Secondly, even with a prospective protocol, two measurement waves only were conducted, and time of exposure was limited. However, structural and organizational changes occurred in all four enterprises under study even during this short observation period, thus reflecting the accelerated dynamics of work-related stress in current economic situation.

Thirdly, as the five indicators of mental health were not independent, some of the reported effects in multivariate analysis may have been overestimated. On the other hand, excluding employees with a respective mental health problem at study onset results in a conservative estimate, as this group may have suffered from previous job stress and that controlling for 'caseness' attenuates the associations under study. A further limitation concerns our decision of categorizing both the predicting and criterion variables, thus loosing information available from continuous data. Linear regressions performed with the normally distributed dependent variables did not yield different results. As this is not a clinical study, the decision of using scores in the upper quartile of the distribution of mental health indicators seems justified. Similarly, using the upper quartile of the ratio between effort and reward as a measure of stressful experience at work is in line with the evidence from several epidemiological studies although analysis of a log-transformed continuous ratio might reveal even stronger effects [25,30,31]. The same remains true with regard to a further test of main effects and interaction terms of the variables 'effort' and 'reward' which formed the basis for the ratio. Studies repeatedly revealed that the effect size of the ratio exceeds the effect sizes of the single variables [8,30,31]. Finally, we cannot exclude a 'healthy worker' effect of the final sample with full data (although this effect would point to a conservative estimate of the observed effect), and we cannot solve the methodological problem of reporting bias as both types of variables, the measure of stressful experience at work and the measures of mental health, were based on self-reported data.

Nevertheless, this study has remarkable strengths. It tests a theoretical model, effort-reward imbalance, that captures some of the core aspects of dynamics of stressful experience at work in a globalized, rapidly changing economy. Moreover, this model was shown to predict a variety of health problems in prospective observational studies in different occupational groups of several countries. Health outcomes include coronary heart disease [25,32], cardiovascular mortality [9] mild-to-moderate psychiatric disorder (mainly affective disorder) [33], alcohol dependence [34], type 2-diabetes [35] and poor self-rated health or poor mental and physical functioning [30,36].

This prospective evidence is supplemented by findings from cross-sectional studies testing associations of effort-reward imbalance at work with mental health, in particular depression [31,37,38]. Having controlled for different socio-demographic and workplace characteristics permits to better identify the evolution of the stress component in a one-year interval and its effect on the worker's mental health. Obviously, the evolution of the stress component in terms of effort-reward imbalance reflects some of the organizational changes in the enterprises.

The validity of reported results is further supported by two observations. First, in a cross-sectional analysis of the baseline data from the Somstress study, effort-reward imbalance at work was found to be associated not only with mental health indicators, but also with sickness absence, an indicator that is less vulnerable to reporting bias [22]. Secondly, the psychometric properties of the Belgian (French) version of the effort-reward imbalance questionnaire were tested in a comparative methodological data analysis of five different European countries. The values of internal consistency, discriminant validity of the scales and goodness of fit of the three model components (effort, reward, overcommitment) were well comparable across the five data sets and met psychometric criteria with satisfactory degree in all samples [20]. Finally, to our knowledge, this is the first report on psychosocial occupational health research with reference to the effort-reward imbalance model that explores the dynamics of stressful experience at work over time in a large sample of male and female employees from enterprises with different degrees of economic stability, using a variety of established mental health indicators.

## Conclusion

In conclusion, continuous experience of stress at work over time and recently emerging job stress experience are both associated with elevated risk of mental health problems. Results underline the importance of studying health effects of a globalized, rapidly changing economy and of developing appropriate measures to reduce the respective burden of disease in working populations.

## Competing interests

The author(s) declare that they have no competing interests.

## Authors' contributions

IG: study design, data collection and manuscript writing

FK: study design, data collection and manuscript writing

YC: manuscript writing

JS: creation of the measurement instrument, manuscript writing

## Pre-publication history

The pre-publication history for this paper can be accessed here:


